# Neural correlates of information gain during search revealed by fMRI

**DOI:** 10.1016/j.isci.2026.117445

**Published:** 2026-09-09

**Authors:** Sakrapee Paisalnan, Yashar Moshfeghi, Frank Pollick

**Affiliations:** 1 Department of Electrical Engineering, Silpakorn University, Nakhon Pathom 73000, Thailand; 2 NeuraSearch Laboratory, University of Strathclyde, Glasgow G1 1XQ, UK; 3 School of Psychology & Neuroscience, University of Glasgow, Glasgow G12 8QQ, UK

**Keywords:** information retrieval, information gain, cognition, fMRI

## Abstract

When confronted with uncertainty, humans engage in information-seeking behavior to gather sufficient information. Identifying the brain regions involved offers insight into the underlying cognitive mechanisms. Using Shannon information theory, we quantified the information contained in the image tiles presented in an fMRI experiment. Nineteen participants judged whether a caption described an image by viewing a sequence of tiles and signaling when it matched. Random-effects ANOVA and representational similarity analysis revealed that regions associated with information gain resulted in two complementary patterns. The first, involving right Ph2 and left hIP5, showed a graded relationship with information gain, consistent with tracking currently available perceptual evidence. The second, involving bilateral PGp with left hIP3 and left FG2, was more sensitive to intermediate information gain, possibly reflecting integrative processes relating accumulated visual information to task-relevant semantic expectations. These findings offer a neurocognitive framework for how information is acquired and evaluated during a sequential search.

## Highlights

• Developed quantified stimuli for an fMRI study of sequential information search
• Brain activity in areas Ph2 and hIP5 scale with information gain in a graded fashion
• Areas PGp, hIP3, and FG2 peak at intermediate gain, reflecting integrative evaluation
• Information acquisition and evaluation rely on distinct neural response profiles

## Graphical abstract

**Figure:**
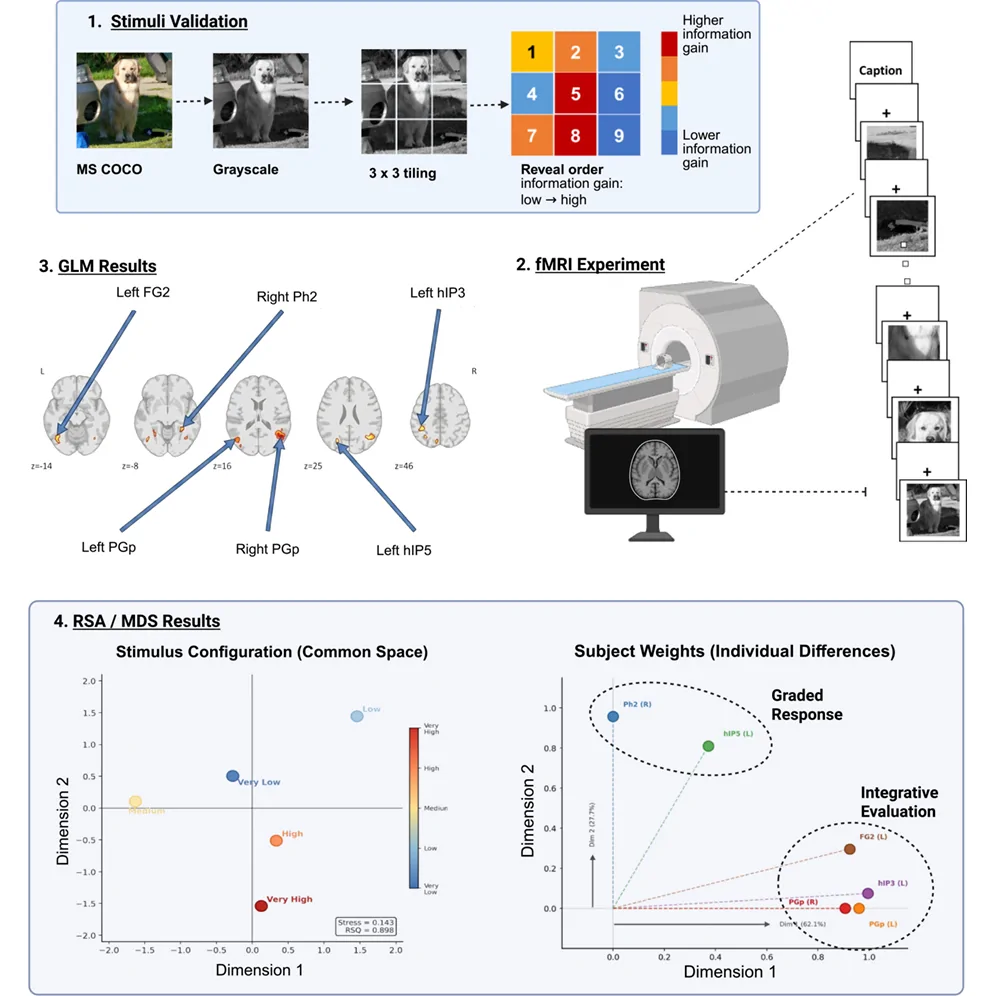

## Teaser

Natural sciences; Biological sciences; Neuroscience; Cognitive neuroscience

## Introduction

Information forms the foundation of our daily activities, underpinning essential tasks, such as knowledge acquisition, decision-making, problem-solving, and communication. During specific tasks, humans often encounter difficulties in completing them. They then recognize a need for information that is missing from their existing knowledge to successfully resolve the task.^1^ Humans inherently engage in a cognitive process known as the information search process (ISP) to seek out information.^2^^,^^3^ The ISP is a multifaceted interplay of thoughts, behaviors, and environmental factors that unfolds in distinct stages. It begins with the recognition of a need, or the encounter with something unclear, leading to a state of uncertainty. To resolve this uncertainty and gain clarity, individuals actively search for relevant information from reliable sources, engaging in an iterative process of gathering and evaluating information. This process continues until the information need is satisfied, marked by a reduction in uncertainty and a sense of fulfillment.^2^ Recent studies associate uncertainty with both the initiation of information search and changes occurring as the search progresses.^4^

Uncertainty plays a pivotal role in the information-seeking process and varies in different contexts and developments.^5^^,^^6^^,^^7^ It often triggers an information need, particularly in unfamiliar tasks. As a strategic response to this uncertainty, individuals seek information to enhance their understanding and satisfy their information needs. Information gain—defined as the reduction in uncertainty achieved through newly acquired information—serves as a critical mediator between uncertainty and satisfaction.^8^ It drives the pursuit of relevant information, reducing uncertainty and increasing knowledge and understanding.^9^^,^^10^^,^^11^^,^^12^ Information gain decreases uncertainty and leads to the satisfaction of an information need. Recent studies of predecisional search extend this view, showing that people adaptively reduce several distinct forms of uncertainty rather than optimizing a single quantity.^13^ This progressive reduction of uncertainty can be understood as an accumulation of evidence toward a decision, a process that fMRI has linked to the moment of recognition.^14^ Through the lens of information theory,^15^ the uncertainty or lack of information a person experiences when seeking answers or clarifications can be understood as entropy. Therefore, information gain represents the reduction of this entropy as relevant information is acquired during the search process. Using information theory, we can quantify the efficiency of a search by measuring how much uncertainty is reduced with each new piece of information.

Brain imaging experiments using fMRI have revealed neural correlates of the information seeking process. For instance, during the search for images that match a caption, judgments of relevance were found to be related to activity in the superior frontal gyrus, inferior parietal lobe, and inferior temporal gyrus.^16^ Examination of a complete search process, broken down into separate phases from start to end, suggested a dynamic pattern of interaction among large scale brain networks, such as the Cingulo-Opercular task control, salience, fronto-parietal task control, and the default mode networks.^17^ A related examination of the search process proposed that the ISP could be broken down into separate components: (1) a successful memory retrieval component, (2) an information flow regulation component, and (3) a high-level perception component.^18^ The realization of an information need was related to brain activity in the posterior cingulate cortex.^19^ Moreover, the pattern of brain activity found was consistent with the attention, breadth and balance (ABBA) model.^20^ Namely, the realization of an information need shifts a narrow internal focus to a broad external focus. Apart from this work, a recent fMRI neuroimaging study shows that posterior parietal cortex encodes the certainty expected after sampling, a key intermediate step in estimating information gain.^21^ These previous studies have revealed much about the neural correlates of information search, highlighting the role of coordinated interaction between internal and external information during search, which has received increasing attention within a broader neuroscience of information seeking.^22^ However, a limitation of these previous findings is that the concept of “information” was loosely defined. In the present work, we use a formal definition of information to more precisely explore how information gain drives the dynamic interaction of neural processes during search.

**Figure 1:**
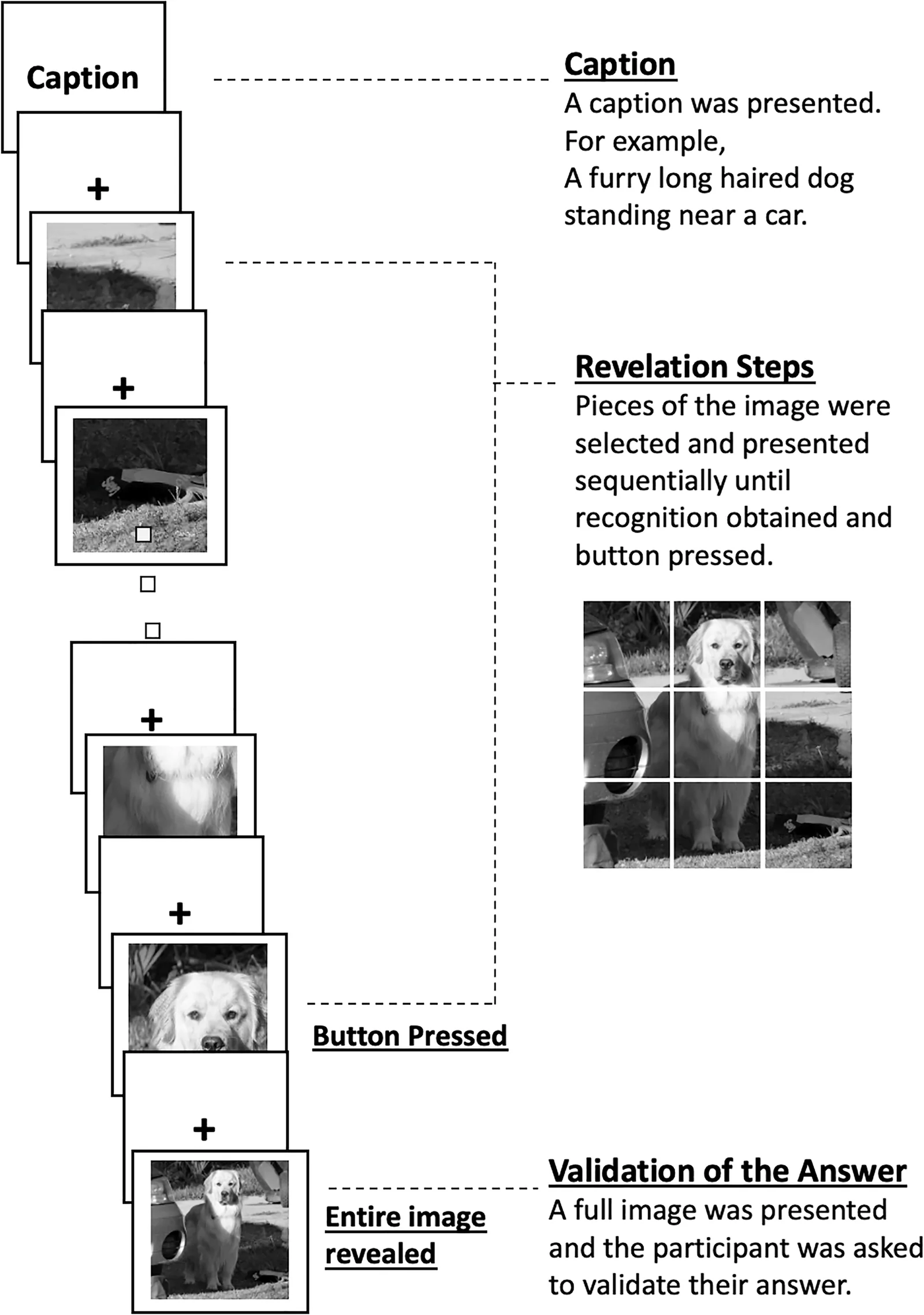
Experimental procedure illustrated as a flowchart with timeline During each trial, participants were asked to judge whether a caption matched an image. A caption was first presented for 4 s, after which image tiles were revealed sequentially (one tile every 2 s), with each presentation separated by a jittered fixation interval of 4–6 s. Participants pressed a button as soon as they recognized that the image matched the caption. The complete image was then presented, and participants were asked to press the button again to confirm that their judgment was correct.

In the present study, we used this formal definition of information gain, along with fMRI brain imaging to deepen our understanding of how information gain influences the brain regions activated during information-seeking behavior. In our experimental task (Figure 1), performed in an fMRI scanner, participants were first presented a text caption, followed by an image presented piecewise by nine image tiles. Participants viewed the image-tile sequence until they could indicate that the image was described by the caption. A measure of information gain (see STAR Methods) used expert assessments of the relevance of each image tile to its corresponding caption. This measure enabled us to quantify the information gain provided by each tile in the image. The image tiles were then presented in a graded sequence, starting with tiles of low information gain and progressing to those with high information gain. Finally, we conducted fMRI data analysis to identify neural correlates associated with the relationship between information gain and information-seeking behavior.

## Results

This study investigated the influence of information gain on human ISPs during an caption-image matching task. A functional magnetic resonance imaging (fMRI) experiment was conducted with 19 participants. In each experimental trial, participants were given a descriptive image caption and then presented sequentially with pieces of an image. They were instructed that when they could determine that the image sequence matched the caption, they should press a button to complete the trial. This process enabled analysis of the neural correlates of information gain throughout the decision-making process.

### Revealing regions of interest in the human ISP

**Figure 2:**
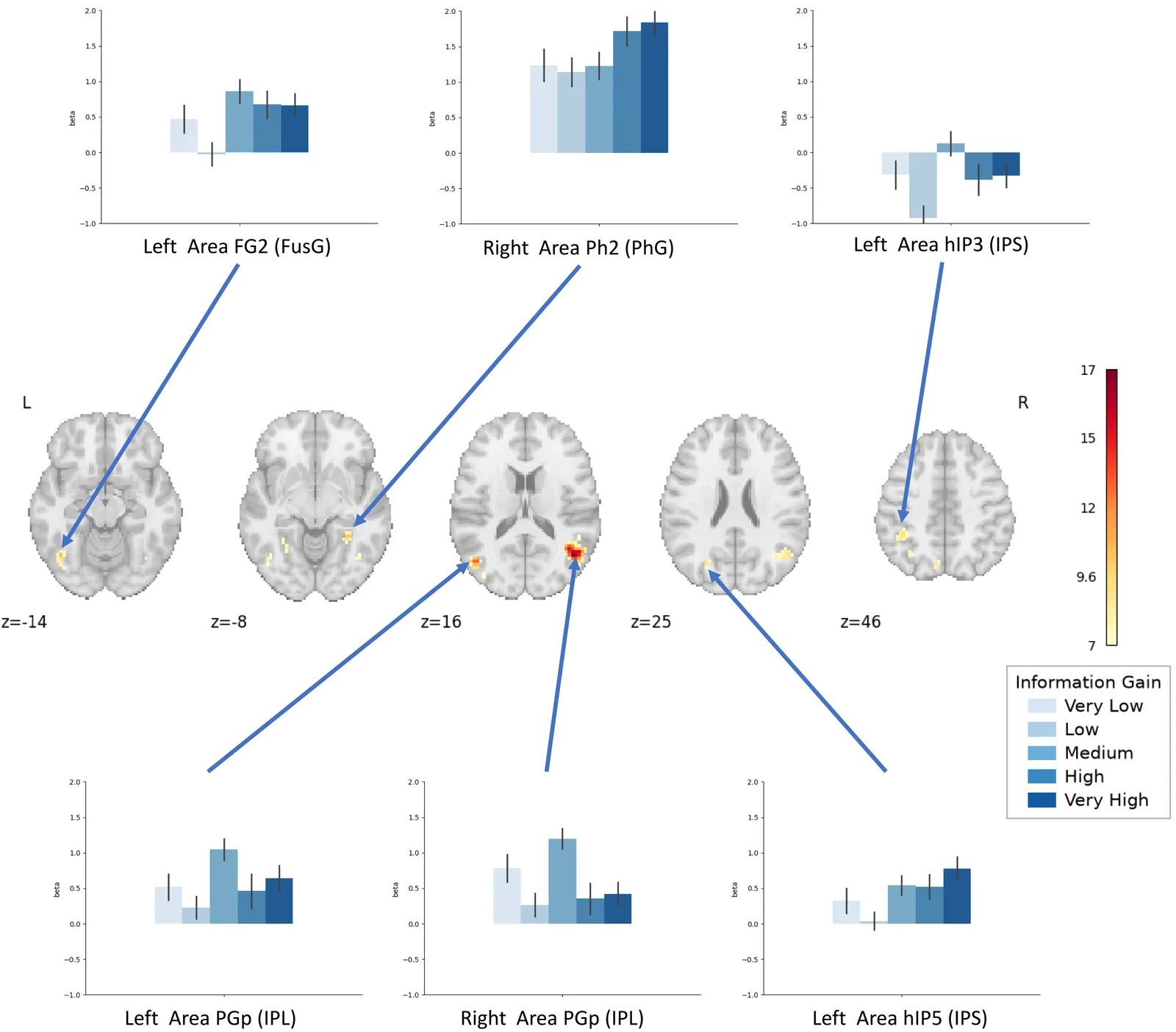
Whole-brain activations associated with the levels of information The six activations are shown along with the beta values for each condition of information gain, projected onto the average anatomical structure across five transverse sections at five different *z* axis values. Bar heights represent mean beta values across participants (*n* = 19) and error bars represent the standard error of the mean (SEM). Activation clusters are thresholded at a voxel-level false discovery rate (FDR) of q < 0.01 with a cluster threshold of 216 mm³ (*p* < 0.05).

Analysis focused on examining brain activity elicited by viewing piecewise presentation of an image and relating this brain activity to a quantitative measure of information gain provided by each piece. A random effects general linear model (GLM) analysis revealed significant neural activations in several key brain regions, each with varying volumes.^23^^,^^24^ The results of the analysis are depicted in Figure 2; Table 1. These visualizations show six distinct clusters exhibiting activation. The right and left PGp (IPL) show highly significant effects, with large volumes of 7673 mm^3^ on the right and 1768 mm^3^ on the left, indicating robust bilateral neural activation. Other regions also exhibit significant effects but with smaller volumes, suggesting more localized neural responses. The right Ph2 (PhG) has a volume of 249 mm^3^, while the left FG2 (FusG) has a volume of 504 mm^3^. These findings indicate specific but less extensive activation patterns compared to the PGp areas. The left hIP5 (IPS) and left hIP3 (IPS) also demonstrate significant neural activity, with volumes of 216 mm^3^ and 916 mm^3^, respectively, suggesting substantial but distinct neural responses.

**Table 1:** Significant clusters from the whole-brain analysis of information gain

Cytoarchitectonic area	Macroanatomical location	Hem.	X	Y	Z	F(4, 72)	*p* value	Voxel *(mm*^*3*^*)*
Area PGp (IPL)	angular gyrus	R	45	‒64	16	17.30	<0.000001	7,673
Area Ph2 (PhG)	temporal fusiform cortex	R	30	‒46	−8	10.32	0.000001	249
Area hIP5 (IPS)	angular gyrus	L	−27	−70	25	8.79	0.000008	216
Area hIP3 (IPS)	postcentral gyrus	L	−39	−43	46	9.04	0.000006	916
Area PGp (IPL)	angular gyrus	L	−48	−70	16	13.11	<0.000001	1,768
Area FG2 (FusG)	temporal occipital fusiform cortex	L	−42	−64	−14	10.63	0.000001	504

Significant clusters from the whole-brain analysis. Anatomical labels follow the Jülich-Brain cytoarchitectonic atlas,^25^ along with macroanatomical location. Coordinates are reported in MNI space (mm).

Hem, hemisphere; L, left; R, right. Coordinates (X, Y, and Z) refer to the peak voxel in MNI152 space.

The beta weights for each level of information were extracted from each activation cluster. The plot of beta weights for all activation clusters is shown in Figure 2. A post hoc analysis was conducted to determine the significance of differences between levels of information across activation clusters (shown in Tables S8, S9, S10, S11, S12, and S13). The results of the post hoc analysis of the activation cluster in the right PGp indicate that the beta weight for the medium level of information differs significantly from those of the low, high, and very high levels of information. This pattern differs slightly from the activation cluster in the left PGp, where the beta weight for the medium level of information is only significantly different from that of the low information level.

Additionally, there are differences in the beta weights of the activation cluster in the left FG2 between the low level of information and the medium, high, and very high levels of information. In the hIP5 cluster, there is a significant difference only between the low and high levels of information. The left hIP3 cluster also shows a significant difference between the low and medium levels of information.

### Investigating the neural correlates of information gain during information search tasks

**Figure 3:**
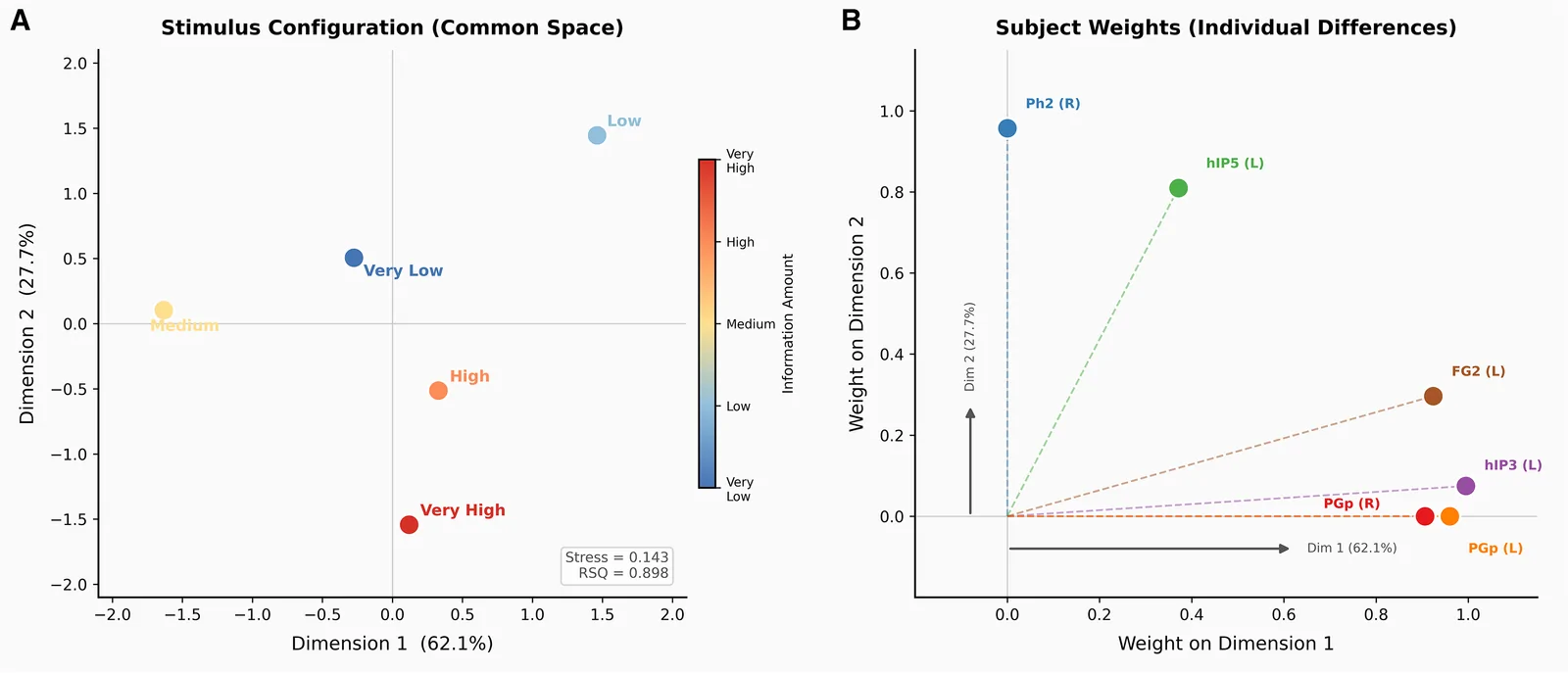
Multidimensional scaling analysis of the levels of information gain Results of the individual-differences multidimensional scaling (INDSCAL) analysis of the five information-gain levels, based on the six brain-activation clusters. (A) The common space plot derived from the multi-dimensional scaling (MDS) analysis, showing the different levels of information gain. The plot reflects the similarities and differences among the levels, as influenced by the weights of the six brain activation clusters. (B) The individual subject weight plot of all six clusters of brain activation where the subject weights indicate the importance of each dimension for the levels of stimulus.

We further investigated the neural correlates of information gain by performing a representational similarity analysis (RSA)^26^ using the activity for each level of information gain for each of the six clusters (see Figure S7 in supplemental information). The RDMs were analyzed using multidimensional scaling (MDS) to find a low dimensional representation of the pattern of brain activity among the six clusters.^27^ The two-dimensional INDSCAL solution was used as it provided an acceptable fit to the dissimilarity data (S-stress = 0.143, RSQ = 0.898; 89.8% of variance accounted for). Adding a third dimension did not meaningfully improve the fit and is not reported further. The results from the MDS analysis include a plot of the two-dimensional common space representation of the five different levels of information gain (Figure 3A) as well as a plot of the subject weights (Figure 3B). The subject weights indicate how much the activity in each cluster influenced dimension 1 and dimension 2 in the common space. Interpretation of the meanings of dimensions 1 and 2 provides us with insight into how the different brain regions represent information gain.

According to the results of the MDS analysis, the common space plot in Figure 3A illustrates that dimension 1 accounts for 62.1% of the overall importance. However, the levels of information amount are not arranged as a monotonic gradient along this dimension; instead, the medium level lies at the most extreme negative position, while the low level lies at the most extreme positive position. The very low, high, and very high levels are located near the middle of the dimension 1 axis. This non-monotonic pattern suggests that dimension 1 does not track the quantity of information available, but may instead reflect processing demands at intermediate levels of information gain. The placement of the medium level at the extreme may represent an ambiguous state that requires more cognitive effort to extract and resolve visual information for the task. In contrast, low information may be processed primarily as isolated visual features with minimal semantic engagement.

Dimension 2 of the common space plot accounts for 27.7% of the overall importance. Unlike dimension 1, dimension 2 was consistent with a nearly monotonic gradient from positive (low and very low) to negative (high and very high), with the medium level positioned near zero. This ordering closely tracks the total amount of information. Consequently, dimension 2 appears to track the overall quantity of visual information available to verify whether the caption matches the full image.

The subject-weights plot (Figure 3B) shows that the two dimensions are driven by distinct groups of activation clusters. Bilateral PGp, left hIP3, and left FG2 contribute predominantly to dimension 1 (weights ≥ 0.91 on dimension 1; ≤ 0.30 on dimension 2). The right Ph2 and left hIP5 contributes predominantly to dimension 2 (weights 0.96 and 0.81 in dimension 2; 0.00 and 0.37 in dimension 1). This subject-weight plot suggests the dimensional dissociation of two functionally distinct neural mechanisms, particularly among the regions contributing to dimension 2, which showed an association with magnitude coding.^28^

## Discussion

This study aimed to simulate an information retrieval scenario within an fMRI environment by asking participants to determine if a caption accurately described an image based on sequentially presented image tiles. This task was designed to mimic the process of gathering information during an information search, which requires the integration of information over time. The study involved 29 right-handed participants with normal or corrected-to-normal vision, and after data preprocessing and quality checks, the data from 19 participants were analyzed. The experiment used a set of stimuli consisting of images divided into nine tiles, with a quantitative measure of the information gain provided by each tile. The study provided insights into the brain’s mechanisms for processing information during search-like tasks, with fMRI data used to identify regions associated with these cognitive processes.

A GLM analysis of brain activity across the different levels of information gain revealed significant activations across several brain regions, particularly the bilateral PGp (inferior parietal lobule, IPL). These activations suggest an important role for bilateral PGp in the integration and evaluation of partially accumulated information during the task. Although the IPL has already been implicated in evidence accumulation and decision-making under uncertainty,^22^^,^^29^ the present pattern of results suggests that bilateral PGp may be more strongly associated with evaluative or integrative processing than with a simple monotonic accumulation of evidence. Further activations were found in the right Ph2, left FG2, and the left hIP5 and hIP3.

To characterize similarities in activation profiles across levels of information gain, RSA was applied to the regional activation patterns derived from the GLM, and the resulting similarity structure was visualized using multidimensional scaling (MDS). Interpretation of the structure of this two-dimensional solution suggested dissociable roles of the brain regions involved. Dimension 1, associated most strongly with bilateral PGp, left hIP3, and left FG2, may reflect integrative evaluation of caption-image consistency, potentially involving contextual interpretation under conditions of partial uncertainty. Dimension 2, associated most strongly with right Ph2 and left hIP5, showed a near-monotonic relationship with information gain and appeared to correspond to the amount of information currently available to guide the decision.

### The bilateral inferior parietal lobule (PGp), left hIP3, and left FG2 and interpretation of dimension 1

Our analysis of the contrast between different conditions of information gain revealed significant clusters in bilateral PGp, a region located in the inferior parietal cortex. The PGp region, which constitutes the posterior part of PG, has been associated with the default mode network.^30^^,^^31^^,^^32^ It has also been suggested to have functional connections with the parahippocampal cortex, forming a route for visuospatial processing in the parietal cortex. This parahippocampal connection is critical for spatial visual processing of scenes related to spatial information.^33^ In addition, PGp has been reported to serve as a hub linking the occipital and parietal cortex for the transformation of visual input to visual association information.^34^

The results from multidimensional scaling (MDS) indicate that the bilateral PGp clusters loaded predominantly on dimension 1 along with left hIP3 and left FG2 (weights ≥ 0.91) but contributed minimally to dimension 2. The beta weights of the bilateral PGp clusters peaked at mid information gain, forming an inverted-U tuning profile. Rather than reflecting a graded sensitivity to the quantity of information alone, this pattern may indicate increased processing demands when available evidence is informative yet insufficient to fully resolve the decision. Participants may therefore engage in greater integrative evaluation when partially accumulated visual information must be related to the developing interpretation implied by the caption. Tiles containing very little information may provide insufficient evidence to strongly engage evaluative processes, whereas highly informative tiles may reduce interpretive demands because the decision becomes comparatively straightforward.

In the present task, bilateral PGp may support the integration of perceptual evidence across sequentially revealed tiles while evaluating its relevance to the contextual or semantic expectations established by the caption. Also, the substantial contribution of left hIP3 and left FG2 on dimension 1 supports a broader interpretation of this dimension as reflecting caption-guided integration under partial uncertainty. Left hIP3, located in the intraparietal sulcus, is involved in visuospatial attention and the integration of object information across spatial locations. Left FG2, located at the junction between early and higher-order visual areas, is associated with categorical visual recognition, visual word processing, and object recognition.^35^^,^^36^ In particular, FG2 has been shown to co-activate with the right intraparietal sulcus, including hIP3, during tasks that involve integration of visual and higher-level semantic information.^35^

In summary, the contribution of bilateral PGp, left hIP3, and left FG2 to dimension 1 suggests that this dimension reflects the cognitive demands associated with evaluating the relevance of accumulated visual evidence to the caption while integrating perceptual information across time.

### Right Ph2 and left hIP5 and interpretation of dimension 2

In contrast to the contribution of four regions to dimension 1, only two regions predominantly contribute to dimension 2: right Ph2 and left hIP5, with weights of 0.96 and 0.81, respectively. Area Ph2, located in the parahippocampal gyrus, is suggested to be involved in scene perception and is associated with the contextual processing of objects and the spatial environment.^33^ Similarly, left hIP5, located in the intraparietal sulcus, is implicated in visuospatial processing and stimulus-related magnitude coding relevant to task goals.^28^

The MDS solution indicated that right Ph2 and left hIP5 contributed strongly to dimension 2, which exhibited a near-monotonic relationship with information level, suggesting a correspondence with the overall quantity of information gain. Accordingly, these findings suggest that these regions may be more strongly associated with tracking currently available information than with the integrative evaluative demands linked to dimension 1. The findings are consistent with the magnitude coding concept, which is associated with the parietal cortex,^28^ whereby neural activity scales with the quantity of incoming information. In this context, right Ph2 may reflect progressively richer scene-level contextual information as increasingly informative tiles are revealed,^37^ while left hIP5 may track the amount of currently available decision-relevant information, potentially contributing to progressive reductions in uncertainty during the caption-image decision.^38^ Taken together, these regions may play complementary roles in tracking visual and contextual evidence currently available for guiding the caption-image decision.^39^

### Relation to information gain with eye movements in natural vision

The present findings may be interpreted within theoretical accounts of natural vision, which propose that eye movements are not passive responses to visual salience but purposive sampling operations deployed to acquire behaviorally relevant information, with gaze strategically allocated to maximize information gain and reduce uncertainty.^7^^,^^11^^,^^40^ Although the current experiment did not involve natural eye movements, the sequential reveal of image tiles similarly required information to be accumulated over time in support of a caption-image decision. Viewed through a natural vision framework, the dissociation between Ph2 and PGp may reflect different temporal scales of information processing during sequential sampling rather than direct analogues of natural eye movement behavior.

Posterior parahippocampal cortex, including Ph2, has been implicated in scene-selective processing, spatial layout coding, and viewpoint-dependent contextual representations tied to the currently viewed environment.^41^^,^^42^ Consistent with its graded relationship to information gain in the present study, Ph2 may therefore preferentially reflect information available within the currently sampled visual evidence, indexing the quantity or contextual richness of information contained in a given perceptual sample as tiles are progressively revealed. By contrast, PGp within posterior angular gyrus/inferior parietal cortex has been linked to multimodal integration, contextual updating, and the combination of perceptual, semantic, and mnemonic information into coherent representations.^43^^,^^44^ Its contribution to dimension 1 is therefore more consistent with a role in integrating information across sequential samples, particularly when interpretation requires relating incoming perceptual evidence to semantic expectations or contextual meaning.

Within this natural vision framework, FG2 and the intraparietal regions hIP3 and hIP5 may support complementary stages of information processing. FG2 has been associated with higher-order visual and category-level representations and may contribute to transforming sampled visual input into increasingly meaningful object or semantic representations,^45^^,^^46^ a role compatible with the proposed integrative contribution of PGp. Likewise, intraparietal cortex has been implicated in visuospatial attention, attentional orienting, and the selection of behaviorally relevant information during visual search,^47^^,^^48^^,^^49^ and functional differentiation among IPS subdivisions has been reported.^50^ Accordingly, the differential contributions of hIP3 and hIP5 in the present analysis may reflect distinct roles of intraparietal cortex in information sampling and evaluation.

In conclusion, our study investigated the neural mechanisms associated with sequential information gathering during a caption-image matching task designed to mimic information acquisition during natural information search in humans. The findings suggested two partially dissociable response patterns involved in information acquisition during search-like behavior: one associated with tracking currently available information and another associated with integrative evaluation of accumulated evidence. The first pattern involved right Ph2 and left hIP5, which showed a graded relationship with information gain and appears consistent with tracking the quantity of perceptual evidence currently available. The second pattern involved bilateral PGp together with left hIP3 and left FG2, which showed sensitivity to intermediate levels of information gain and may reflect caption-guided integration and evaluation of accumulated visual information under partial uncertainty.

These findings suggest that information gathering during search-like behavior may involve a dynamic interaction between mechanisms that track currently available evidence and mechanisms that integrate successive information samples into coherent, goal-relevant interpretations. More broadly, the results provide a neurocognitive framework for understanding how information is progressively acquired and evaluated during sequential search.

### Limitations of the study

A limitation of the present study is the relatively small sample size of 19 analyzed participants. Ten participants were excluded from a sample of twenty-nine recruited for this experiment due to pre-established quality-control criteria (excessive head motion > 3 mm or 3°, failure to follow instructions, or below-chance performance). One possible contributing factor to participant exclusion was the relatively high task burden associated with the experiment, as trial duration could become extended when a greater number of tiles were revealed, potentially increasing cognitive fatigue and reducing compliance or performance over prolonged testing. Although the analyzed sample size is comparable to that of other event-related fMRI studies examining complex cognitive tasks with a high number of trials, future work conducting the experiment with larger samples and a higher number of trials would be valuable to confirm the generalizability of the present findings and to increase the sensitivity to detect smaller effect sizes.

Additionally, visual complexity may co-vary with information gain in the experimental stimulus set. Information gain may be defined relative to the caption. Therefore, a tile that is visually complex and irrelevant to the caption may receive a low information gain value. So, the two properties were not orthogonally manipulated. Future studies focusing on independently varying visual complexity and information relevance would help to isolate their respective contributions to the observed neural responses.

Also, variability in the number of tiles viewed before responding may reflect individual differences in response thresholds. Although a self-paced design is central to simulating naturalistic information search, future studies could complement this approach with fixed-presentation conditions to help disentangle these factors.

## Resource availability

### Lead contact

Requests for further information and resources should be directed to and will be fulfilled by the lead contact, Sakrapee Paisalnan (paisalnan_s@su.ac.th).

### Materials availability

This study did not generate new unique reagents.

### Data and code availability

• Representational dissimilarity matrices and statistical probability maps have been deposited at OSF and are publicly available as of the date of publication; the DOI is listed in the key resources table (https://doi.org/10.17605/OSF.IO/F9HSB). The caption–image stimulus set generated in this study is available from the lead contact upon request. Raw fMRI data cannot be deposited in a public repository because of ethics and participant-consent restrictions.
• This paper does not report original code.
• Any additional information required to reanalyze the data reported in this paper is available from the lead contact upon request.

## Acknowledgments

This work was partially supported by the Department of Electrical Engineering, Faculty of Engineering and Industrial Technology, 10.13039/501100006436Silpakorn University.

## Author contributions

Conceptualization, S.P., Y.M., and F.P.; methodology, S.P., Y.M., and F.P.; investigation, S.P.; formal analysis, S.P., Y.M., and F.P.; data curation, S.P. and F.P.; visualization, S.P.; writing – original draft, S.P., Y.M., and F.P.; writing – review and editing, S.P., Y.M., and F.P.; funding acquisition, S.P.; resources, F.P.; supervision, Y.M. and F.P.

## Declaration of interests

The authors declare no competing interests.

## STAR★Methods

### Key resources table

**Table:** 

REAGENT or RESOURCE	SOURCE	IDENTIFIER
**Deposited data**		

RDMs and statistical maps	OSF	https://doi.org/10.17605/OSF.IO/F9HSB
Stimulus set	This paper	Available from lead contact upon request

**Software and algorithms**		

BrainVoyager 20 and 21.4	Brain Innovation	https://brainvoyager.com/
IBM SPSS Statistics v29	IBM	https://www.ibm.com/products/spss
siibra v0.4	Forschungszentrum Juelich GmbH	https://fzj-inm1-bda.github.io/siibra/
MS COCO dataset	Lin et al.^51^	https://cocodataset.org/
Jülich-Brain Atlas	Amunts et al.^25^	https://julich-brain-atlas.de/

### Experimental model and study participant details

The experiment examined how the gradual accumulation of visual information influences neural activity. Participants viewed images that were progressively revealed as a sequence of tiles while judging whether a previously presented caption accurately described the image. This task emulated an information retrieval process within the context of functional magnetic resonance imaging (fMRI) scanning. Participants completed a caption–image matching task during fMRI scanning. Each trial began with the presentation of a caption, followed by a sequence of image tiles. Participants indicated as soon as they judged that the tiles matched the caption. The complete image was then displayed, and participants confirmed whether their decision was correct. The independent variable was the information level associated with each image tile, and the dependent variable was the corresponding Blood-Oxygen-Level-Dependent (BOLD) response measured with fMRI.

All participants recruited in this study were healthy adult human volunteers; no animal models, cell lines, primary cell cultures, plant, or microbial strains were used in this study, and cell-line authentication and mycoplasma testing are therefore not applicable. Participant sex was recorded (see Participants below); gender, ancestry, race, and ethnicity were not collected. The study was not designed or statistically powered to evaluate the influence of sex or gender on the outcome measures, and no sex- or gender-based analyses were performed; this is acknowledged as a limitation of the present study. The study followed a within-participant design where the participants were asked to engage in the information-search task within the scanner and were exposed to information-gain conditions, so there was no allocation of participants to separate experimental groups. All procedures were approved by the Ethics Committee of the College of Science and Engineering, University of Glasgow (Application No. 300180236), and all participants provided written informed consent before participating in the experiment (see also Procedure).

#### Participants

The study included 29 healthy participants (16 females, 13 males), all of whom were right-handed and had normal or corrected-to-normal vision. Most participants were aged 18–23 years (51.72%), followed by 24–29 years (31.03%). The majority were students (89.66%), with smaller numbers of unemployed participants (6.90%) and researchers (3.45%). Most participants were native English speakers (58.62%), while 37.93% reported advanced English proficiency. Participants reported an average of approximately 14 years of online search experience, primarily using search engines for web browsing, with video and image searches being secondary in frequency.

Ten participants were excluded according to predefined data quality criteria. Seven were excluded because head motion exceeded 3 mm or 3°, resulting in excessive motion-related artifacts. Two participants were excluded because they failed to follow the task instructions, and one was excluded because task accuracy on identifying captions was below 50%. The final analysis therefore included 19 participants.

### Method details

#### Stimulus selection and validation

One hundred images were selected from the Microsoft Common Objects in Context (MS COCO) dataset.^51^ Images were required to depict non-iconic everyday scenes containing at least two distinct objects, with captions encompassing multiple objects rather than a single localized region. All images were converted to grayscale and divided into a 3 × 3 grid, producing nine image tiles.

A preliminary behavioral study involving 25 participants was conducted to estimate stimulus difficulty. Participants viewed a caption followed by image tiles presented in random order and indicated when they judged that a tile matched the caption. Behavioral responses were used to classify images as easy, moderate, or difficult. Images of moderate difficulty were selected for the fMRI experiment to encourage recognition after approximately four to five tiles.

For each selected image, tile presentation order was determined from least to most relevant to the caption. Relevance was independently rated by three assessors using a 3-point scale (0 = not relevant, 1 = somewhat relevant, 2 = very relevant). Fleiss’ Kappa (*κ*) was used to assess inter-rater agreement, and images with consistent relevance ratings were retained. The final stimulus set comprised 24 images, the majority of which were of moderate difficulty. To maintain participant engagement, two trials contained non-matching caption-image pairs requiring all nine tiles to be viewed, and two trials began with a highly informative first tile to prevent participants from adopting a fixed expectation that early tiles would always contain little information.

#### Calculating information gain of an image tile

We utilized two sources of annotation in order to quantify the information gain associated with each tile. First, the objects present within each tile were already annotated in the MS COCO dataset and were verified by a single experienced annotator, who confirmed the consistency of these object annotations across the nine tiles of every image. However, relevance was annotated by three experienced assessors using a 3-point scale (0 = not relevant, 1 = somewhat relevant, 2 = very relevant), as described in Section Stimulus selection and validation above. These annotations were compiled into two sets, denoted (*O* = *o*_*i*_), representing the objects identified within each tile, and (*R* = *r*_*i*_), representing the relevance levels of these objects with respect to the caption.

The number of objects appearing in a tile ranged from zero to seven across the stimulus set. The combination of three assessors providing 3-point relevance ratings (0 = not relevant, 1 = somewhat relevant, 2 = very relevant) yielded combined scores ranging from 0 (all three rated not relevant) to 6 (all three rated very relevant), with seven possible unique values. Table S1 in the supplemental information summarizes the joint distribution of object counts and aggregate relevance levels across the dataset.

We quantified information within each tile using the Shannon self-information formulation, in which the surprise (*S*_*i*_) associated with an event (i) of probability (*P*_*i*_) is defined as

(Equation 1) $$S_{i}=-\log\left(P_{i}\right)$$

For two events (j) and (k) where (k) is contingent upon (j), the surprise of their conjunction is

(Equation 2) $$S_{\left(j\wedge k\right)}=-\log\left(P_{\left(j\wedge k\right)}\right)=-\log\left(P_{j},P_{\left(k|j\right)}\right)=S_{j}-\log\left(P_{\left(k|j\right)}\right)$$

Applied to our stimuli, we treated the presence of objects in a tile and the relevance of those objects as conditionally related events, since the relevance of a tile’s content is constrained by which objects appear in it. The surprise associated with the conjunction of object presence and relevance, *S*_(obj∧rel)_, was therefore computed as

(Equation 3) $$S_{\left(\text{obj}\wedge \text{rel}\right)}=S_{\text{obj}}-\log\left(P_{\left(\text{rel}|\text{obj}\right)}\right)$$

The probability terms *P*_obj_ and *P*_(rel∣obj)_ were empirically estimated as relative frequencies per-image, calculated across the nine tiles that make up each 3 × 3 image grid. Specifically, *P*_obj_ was taken as the marginal frequency of a given object count among the nine tiles of the image, and *P*_(rel∣obj)_ as the conditional frequency of a given aggregate relevance level given that object count, again within the same set of nine tiles.

This per-image estimation reflects the experimental framing of the paradigm. During a trial, the participant evaluates one specific part of the image against the caption shown at the beginning of the trial, judging whether the caption can adequately represent the entire image. A tile is therefore “surprising,” in the information-gain sense, relative to the other tiles of the same image rather than to tiles pooled across all 24 images—that is, a tile is informative to the observer to the extent that its content is unexpected given the rest of the image. We therefore calculated the information gain separately for each image, since our theoretical interest is in how tiles differ within an image, not in how images differ from one another. A similar local, stimulus-conditioned formulation of surprise underpins prior work on information-driven attention and gaze allocation.^11^^,^^52^

The information level was calculated from both inputs described above: the number of objects present in each tile (0–7) and the aggregate relevance level (0–6, derived from three assessors on a 3-point scale). The resulting values took 13 distinct values across the stimulus set. To define regressors for the fMRI general linear model, these values were assigned to five ordinal levels as follows Very Low, Low, Medium, High, and Very High as using equal-frequency binning. This yielded approximately equal numbers of tile observations per level: Very Low (*n* = 474), Low (*n* = 726), Medium (*n* = 471), High (*n* = 310), and Very High (*n* = 377); total *N* = 2,358. Equal-frequency binning was chosen to ensure balanced trial counts across conditions for stable first-level GLM estimation.

#### Task

The experimental task was designed to examine how participants accumulated visual information over time while making a caption--image decision. Each trial began with the presentation of a caption, followed by a sequence of image tiles sampled from the corresponding image. Participants indicated as soon as they judged that sufficient information had been acquired to determine that the caption correctly described the image. This sequential accumulation of visual information provides a controlled analogue of information acquisition during search while remaining compatible with event-related fMRI.

Each image was divided into nine tiles, which were presented individually in a predetermined order (see stimulus selection and validation). Information accumulated progressively as additional tiles were revealed, allowing participants to integrate evidence over time before making their decision. Individual tiles varied in their relevance to the caption and therefore differed in the amount of information they provided.

Each trial began with presentation of the caption for 4s. Image tiles were then presented sequentially for 2s each, separated by a jittered fixation interval of 4–6s. Participants pressed a response button as soon as they judged that the caption matched the image. The complete image was then displayed for 2s, after which participants pressed the response button again if the response they had provided was correct.

#### Procedure

Ethical approval (Application No. 300180236) was obtained from the Ethics Committee of the College of Science and Engineering, University of Glasgow. All participants provided written informed consent and were informed that they could withdraw from the study at any time without penalty. Participants received compensation at a rate of £6/h.

Before scanning, participants completed MRI safety screening and changed into metal-free clothing to minimize imaging artifacts. They then completed a questionnaire assessing demographic characteristics and online search experience, followed by a set of practice trials to familiarize themselves with the task.

Participants subsequently completed the main experimental task during fMRI scanning. The scanning session lasted approximately 1 h, including around 50 min of functional imaging and 10 min of structural image acquisition. During the task, participants completed 24 caption-image trials involving information acquisition and response validation. Following the scan, participants completed a brief exit questionnaire describing their experience of the experiment.

#### Image acquisition

Imaging was performed using a 3T Siemens TIM Trio MRI scanning at the Center for Cognitive Neuroimaging, University of Glasgow. Functional volumes were acquired using a T2∗-weighted gradient echo, echo-planar imaging sequence (68 interleaved slices, TR: 2000 ms, TE: 30 ms, voxel size: 3 × 3 × 3 mm, FOV: 210 mm, matrix size: 70 × 70). Also, a high-resolution anatomical volume was acquired at the end of the scanning using a T1-weighted sequence (192 slices, TR: 1900 ms, TE: 2.52 ms, voxel size: 1 × 1 × 1 mm, FOV: 210 mm, matrix size: 256 × 256).

#### fMRI preprocessing

Functional MRI data were analyzed using BrainVoyager 20.^53^ Preprocessing followed a standard pipeline and included slice-timing correction (trilinear interpolation), three-dimensional motion correction using rigid-body alignment to the first functional volume, removal of linear trends, and high-pass temporal filtering (0.0025 Hz) to reduce low-frequency physiological noise. Functional images were co-registered to each participant’s anatomical image and normalized to Montreal Neurological Institute (MNI) space. Finally, the normalized functional images were spatially smoothed with a 6 mm full-width at half-maximum (FWHM) Gaussian kernel prior to group analysis.

### Quantification and statistical analysis

#### General Linear Model (GLM) analysis

To identify brain regions associated with information gain, first-level analyses were performed separately for each participant using a voxel-wise General Linear Model (GLM).^23^^,^^24^ The GLM included regressors modeling image-tile presentation at each of the five information gain levels together with head-motion correction parameters. Predictor time courses were convolved with a hemodynamic response function. Individual contrast estimates were entered into a second-level random-effects analysis of variance with information gain as a within-participant factor.

Whole-brain statistical maps were thresholded using a voxel-wise false discovery rate (FDR; *q* < 0.01). Significant clusters were subsequently identified using cluster-level correction based on 1,000 Monte Carlo simulations, with image smoothness estimated at FWHM = 2.474 functional voxels. The resulting minimum cluster size threshold was 216 mm^3^, corresponding to *p* < 0.05.

Peak MNI coordinates from significant clusters were anatomically labeled using the Harvard--Oxford Cortical and Subcortical probabilistic atlases (25% probability threshold; 2 mm resolution) implemented in nilearn (v0.13). More detailed cytoarchitectonic labels were obtained from the Jülich-Brain Atlas^25^ using siibra (v0.4). For each coordinate, the highest-probability non-boundary region was selected as the primary anatomical label.

For each significant cluster, beta weights were extracted at each of the five information gain levels for visualization and post hoc analysis. Pairwise comparisons between information gain levels were performed using Tukey’s honestly significant difference (HSD) test in SPSS Statistics (v29.0.2.0).

#### Representational similarity analysis

Representational similarity analysis (RSA) was used to characterize neural representations associated with different levels of information gain.^26^ To generate the activation patterns for RSA, a separate GLM was estimated for each participant. The model included regressors representing image-tile presentation at each of the five information gain levels, fixation at the beginning of the trial, and head-motion parameters. Predictor time courses were convolved with a hemodynamic response function, and second-level random-effects ANOVA was performed with information gain as a within-participant factor. Contrasts against fixation produced five whole-brain statistical maps, one for each information gain level (very low, low, medium, high, and very high).

Spherical regions of interest (ROIs; 4 mm radius) were centered on the peak coordinates of the six activation clusters identified in the primary GLM analysis. First-level RSA was performed in BrainVoyager 21.4 using Euclidean distance to quantify pairwise dissimilarities between activation patterns for each information gain condition. This yielded one representational dissimilarity matrix (RDM) for each ROI.

Multidimensional scaling (MDS) was then performed using the ALSCAL procedure^27^ with the INDSCAL model in IBM SPSS Statistics (v29.0.2.0). The six RDMs served as input to derive a common representational space while estimating region-specific dimension weights. INDSCAL is suited for this analysis because it derives a common stimulus space shared across brain regions and provides region-specific weight vectors that quantify how strongly each brain region relies on each dimension. Two- and three-dimensional solutions were evaluated using Young’s S-Stress and the squared correlation between distances and disparities (RSQ). Although a three-dimensional solution was explored, it was not retained because SPSS indicated potential overfitting (33 parameters estimated from 60 observations), and the third dimension contributed only approximately 10% of the total variance. The two-dimensional solution was therefore selected on the basis of model fit and interpretability.
