# Supplementary material for: Neural correlates of information gain during search revealed by fMRI
Source: iScience. 2026 Sep 9;29(9):117445. doi: 10.1016/j.isci.2026.117445 (PMC13581990; doi:10.1016/j.isci.2026.117445)
Supplement: Supplementary file 1 [file mmc1.pdf]

iScience, Volume *29*

Supplemental information

Neural correlates of information gain during search revealed by fMRI

Sakrapee Paisalnan, Yashar Moshfeghi, and Frank Pollick

Figure S1: Cytoarchitectonic localization of the first activation cluster, overlaid on the right Temporal-to-Parietal (GapMap) region.

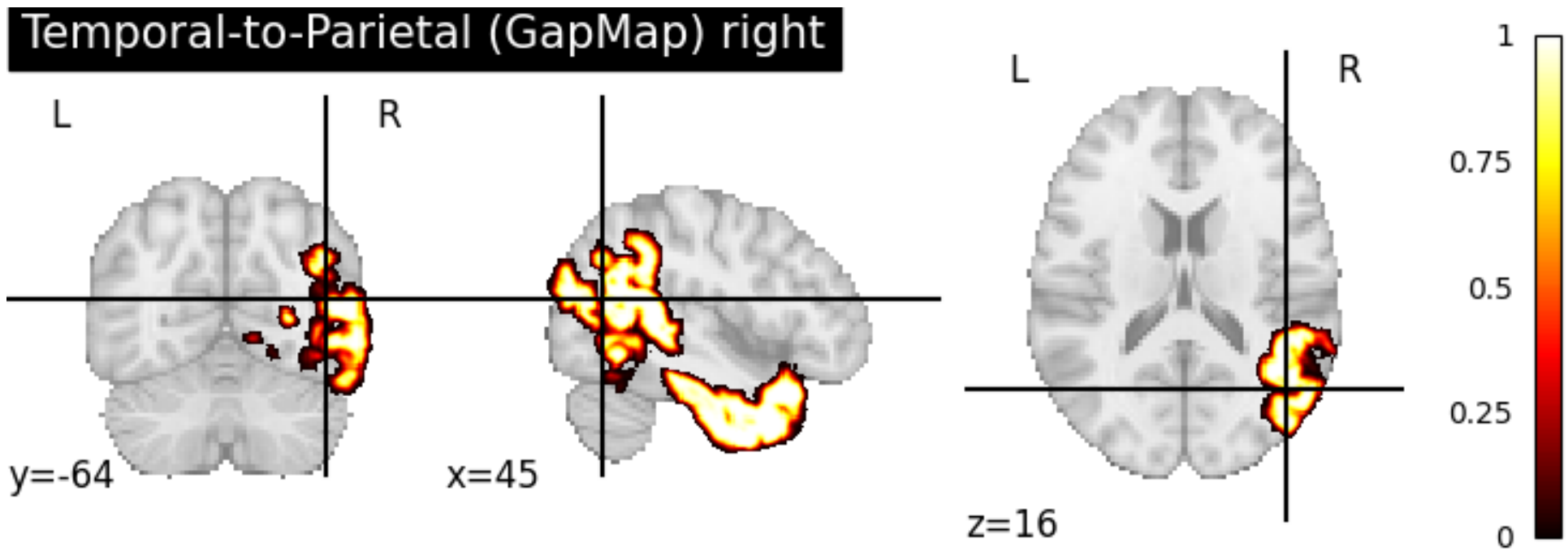

Figure S2: Cytoarchitectonic localization of the second activation cluster in right Area Ph2 (PhG).

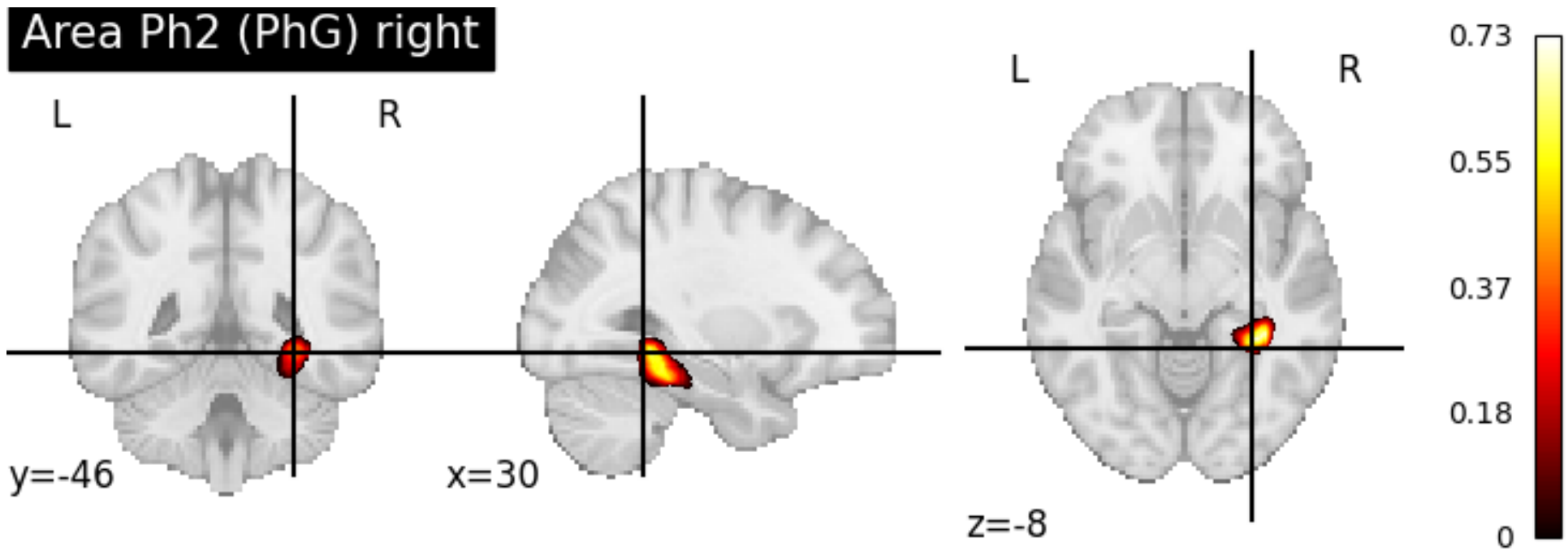

Figure S3: Cytoarchitectonic localization of the third activation cluster in left Area hIP5 (IPS).

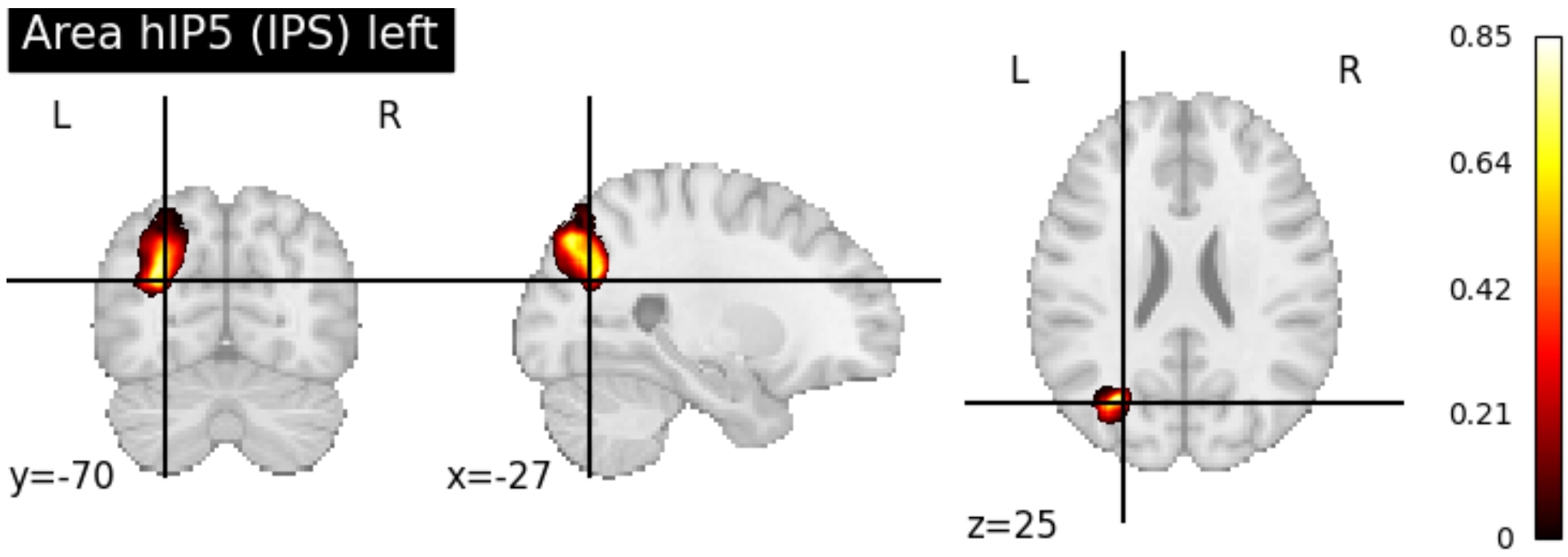

Figure S4: Cytoarchitectonic localization of the fourth activation cluster in left Area hIP3 (IPS).

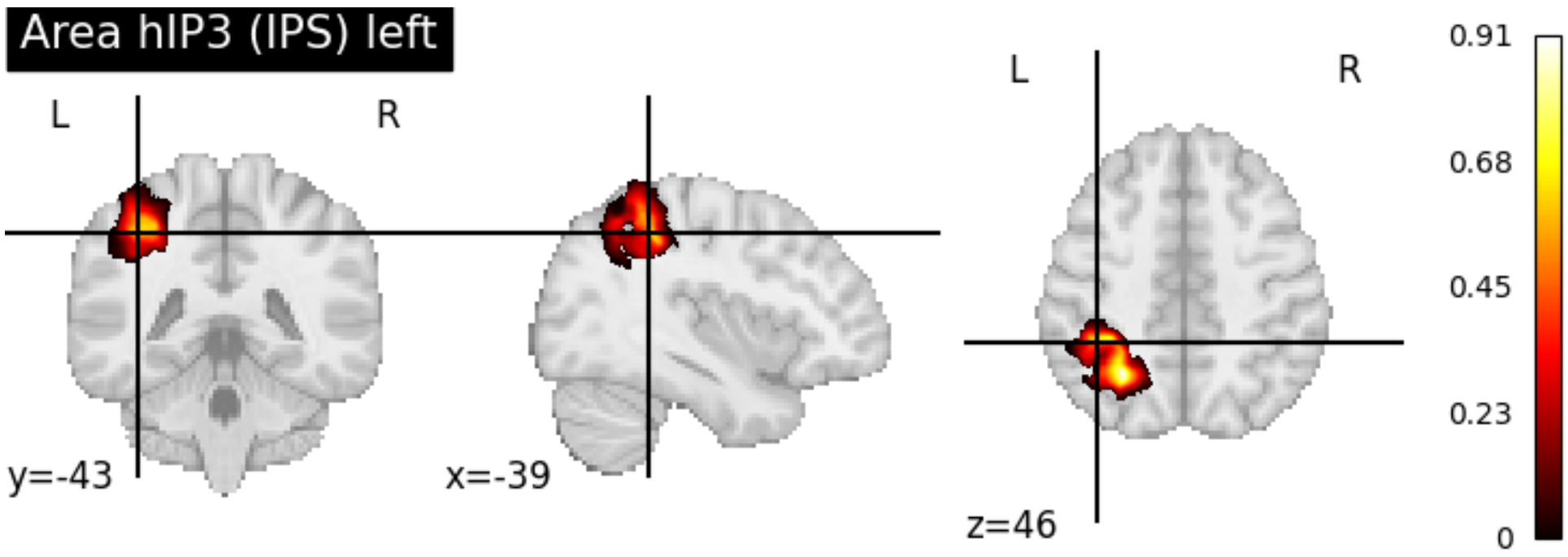

Figure S5: Cytoarchitectonic localization of the fifth activation cluster in left Area PGp (IPL).

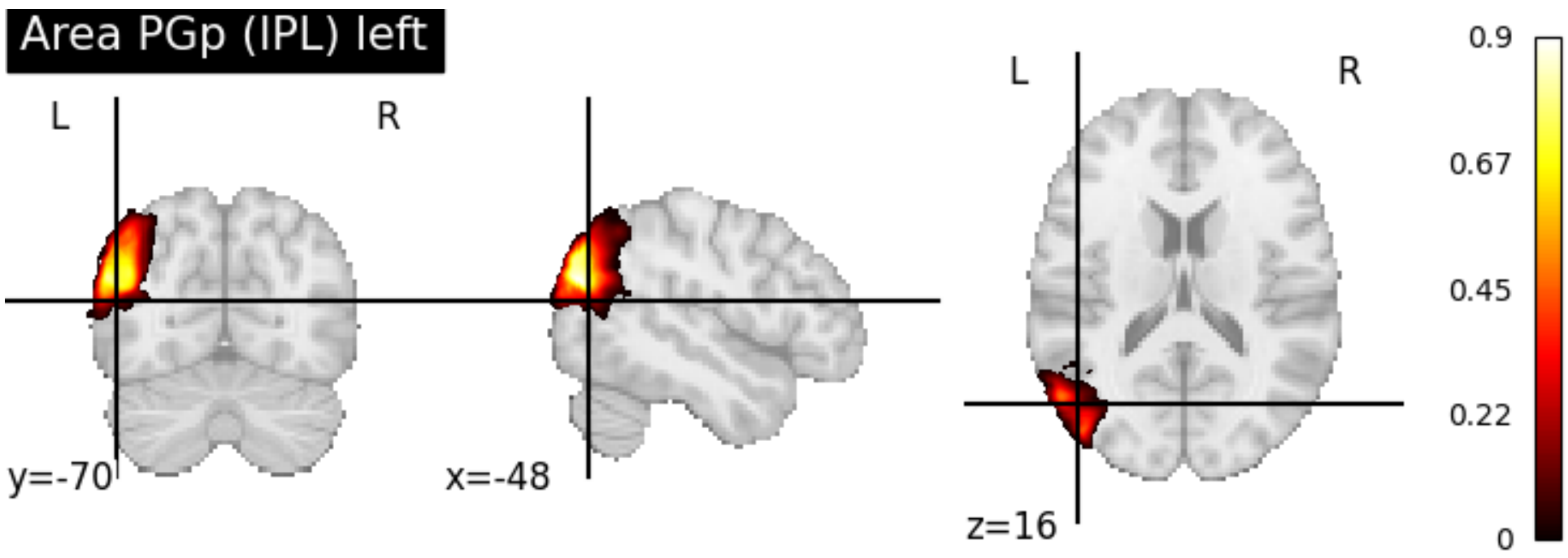

Figure S6: Cytoarchitectonic localization of the sixth activation cluster in left Area FG2 (FusG).

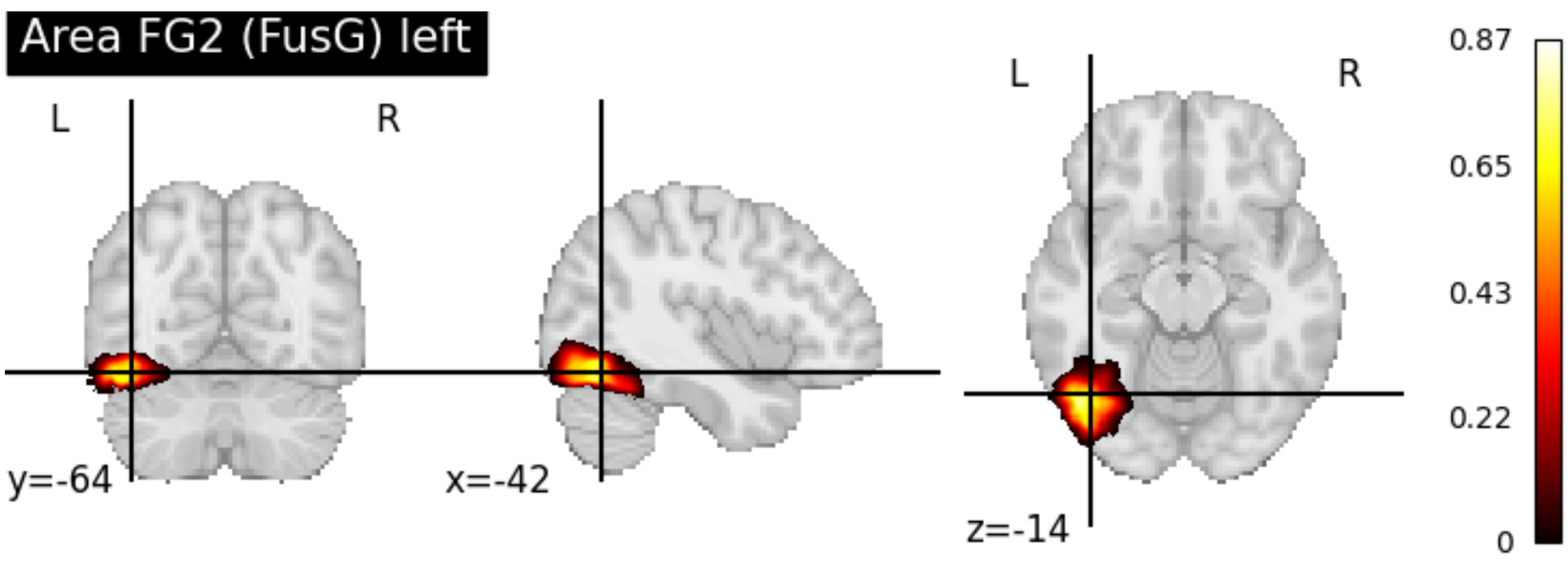

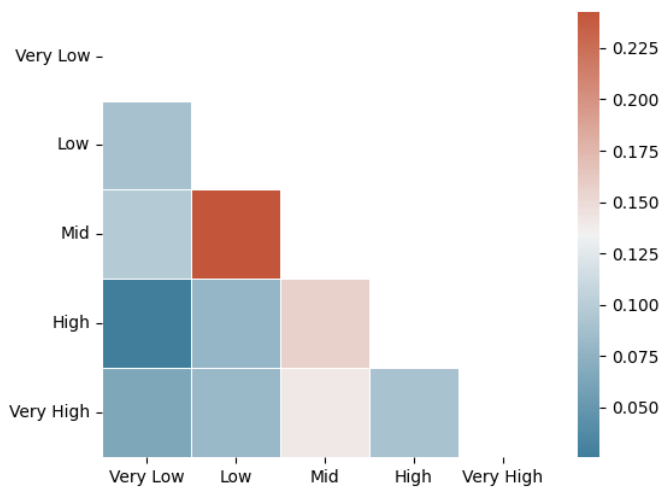

(a) Right Area PGp (IPL)

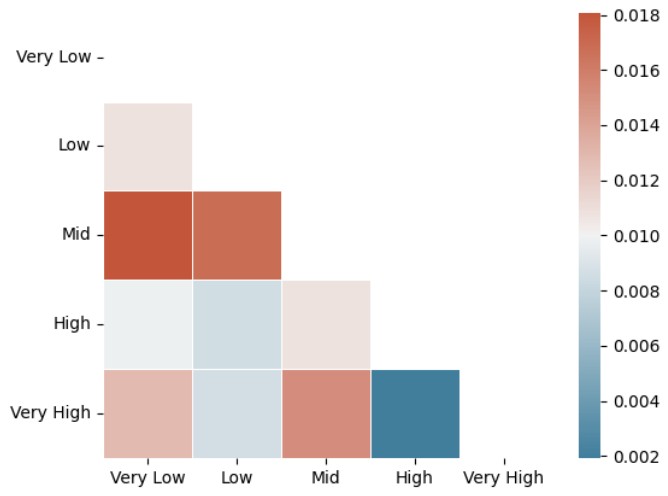

(b) Right Area Ph2 (PhG)

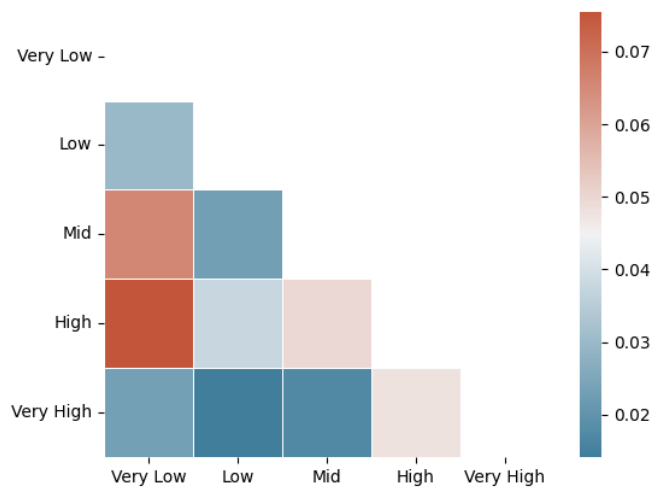

(c) Left Area hIP5 (IPS)

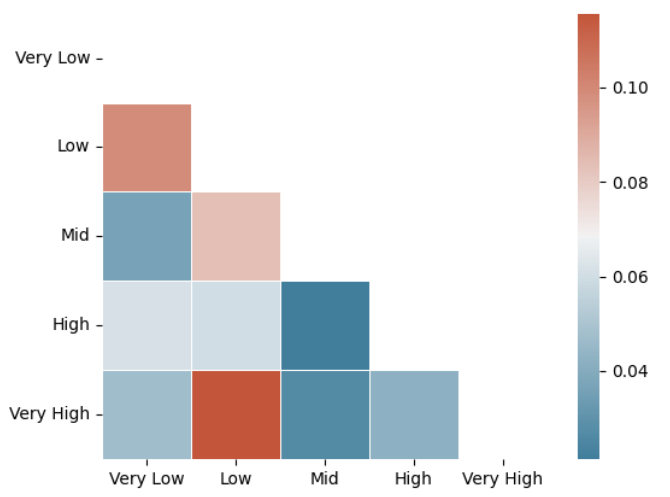

(d) Left Area hIP3 (IPS)

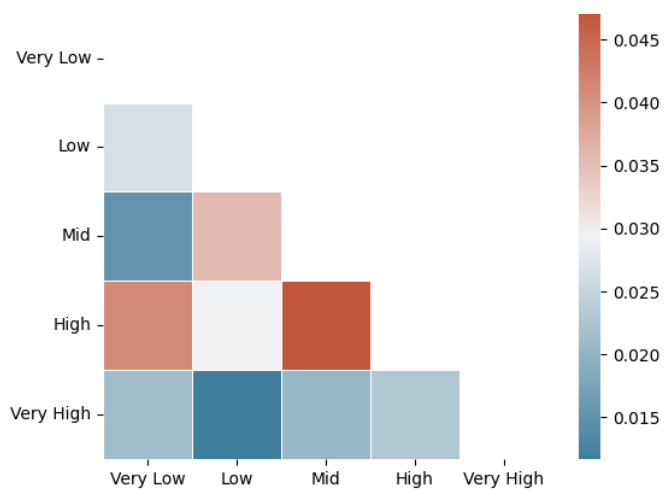

(e) Left Area PGp (IPL)

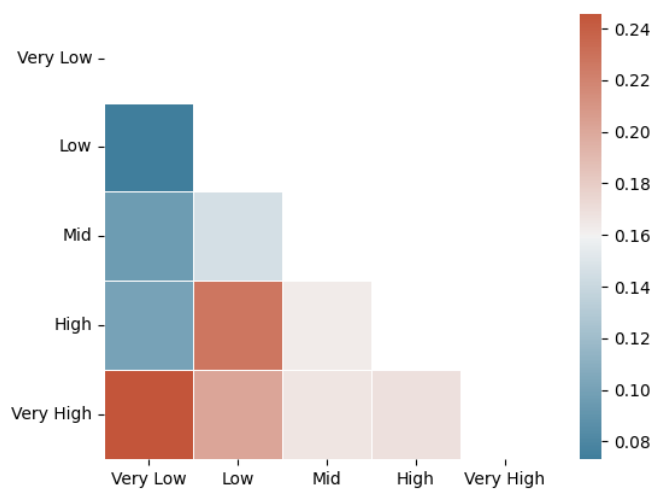

(f) Left Area FG2 (FusG)

Figure S7: Representational dissimilarity matrices (RDM) of the clusters resulting from the general linear model (GLM) analysis: (a) Right Area PGp (IPL), (b) Right Area Ph2 (PhG), (c) Left Area hIP5 (IPS), (d) Left Area hIP3 (IPS), (e) Left Area PGp (IPL), and (f) Left Area FG2 (FusG).

Table S1: The table of relevance judgments and number of objects visible for each tile.

**No. of Objects**	**Relevance**								
	1	2	3	4	5	6	7	8	9
0	0	7	24	0	0	0	0	0	0
1	0	14	8	36	0	31	0	0	0
2	0	3	2	10	0	23	3	1	0
3	0	2	1	11	0	21	6	1	0
4	0	0	0	3	0	2	3	0	0
5	0	0	0	1	0	1	1	0	0
6	0	0	0	0	0	0	0	0	0
7	0	0	0	0	0	0	0	1	0

Table S2: Results from identifying an anatomic label of the first cluster using Julich Brain Atlas and siibra-python including centroid, volume, anatomic label, and map value of each candidate.

centroid	volume	region	map value
(45.0, -64.0, 16.0)	241	Temporal-to-Parietal (GapMap) right	0.59048
(45.0, -64.0, 16.0)	61	Area PGp (IPL) right	0.0199815

Table S3: Results from identifying an anatomic label of the second cluster using Julich Brain Atlas and siibra-python including centroid, volume, anatomic label, and map value of each candidate.

centroid	volume	region	map value
(30.0, -46.0, -8.0)	225	Area Ph2 (PhG) right	0.367754
(30.0, -46.0, -8.0)	223	Area Ph1 (PhG) right	0.338575
(30.0, -46.0, -8.0)	229	Area FG3 (FusG) right	0.293672
(30.0, -46.0, -8.0)	241	Temporal-to-Parietal (GapMap) right	0.19733

Table S4: Results from identifying an anatomic label of the third cluster using Julich Brain Atlas and siibra-python including centroid, volume, anatomic label, and map value of each candidate.

centroid	volume	region	map value
(-27.0,-70.0,25.0)	76	Area hIP5 (IPS) left	0.495289
(-27.0,-70.0,25.0)	68	Area hIP7 (IPS) left	0.220471
(-27.0,-70.0,25.0)	80	Area hIP8 (IPS) left	0.16423
(-27.0,-70.0,25.0)	74	Area hIP4 (IPS) left	0.089669

Table S5: Results from identifying an anatomic label of the fourth cluster using Julich Brain Atlas and siibra-python including centroid, volume, anatomic label, and map value of each candidate.

centroid	volume	region	map value
(-39.0,-43.0,46.0)	70	Area hIP3 (IPS) left	0.587316
(-39.0,-43.0,46.0)	66	Area hIP1 (IPS) left	0.223579
(-39.0,-43.0,46.0)	24	Area 7PC (SPL) left	0.152938
(-39.0,-43.0,46.0)	20	Area 5L (SPL) left	0.0283452
(-39.0,-43.0,46.0)	46	Area 2 (PostCS) left	0.00782226

Table S6: Results from identifying an anatomic label of the fifth cluster using Julich Brain Atlas and siibra-python including centroid, volume, anatomic label, and map value of each candidate.

centroid	volume	region	map value
(-48.0,-70.0,16.0)	60	Area PGp (IPL) left	0.221024
(-48.0,-70.0,16.0)	240	Temporal-to-Parietal (GapMap) left	0.183982
(-48.0,-70.0,16.0)	100	Area hOc4la (LOC) left	0.0916442

Table S7: Results from identifying an anatomic label of the sixth cluster using Julich Brain Atlas and siibra-python including centroid, volume, anatomic label, and map value of each candidate.

centroid	volume	region	map value
(-42.0,-64.0,-14.0)	226	Area FG2 (FusG) left	0.636434
(-42.0,-64.0,-14.0)	232	Area FG4 (FusG) left	0.30901
(-42.0,-64.0,-14.0)	230	Area FG1 (FusG) left	0.0545565

(I) Information Level	(J) Information Level	Mean Difference (I-J)	Std. Error	Sig.	95% Confidence Interval	
					Lower Bound	Upper Bound
1	2	0.5220	0.2521	0.242	-0.1799	1.2239
	3	-0.4139	0.2521	0.475	-1.1157	0.2880
	4	0.4324	0.2521	0.430	-0.2695	1.1343
	5	0.3662	0.2521	0.596	-0.3357	1.0681
2	1	-0.5220	0.2521	0.242	-1.2239	0.1799
	3	-0.9359	0.2521	0.003	-1.6377	-0.2340
	4	-0.0896	0.2521	0.997	-0.7915	0.6123
	5	-0.1558	0.2521	0.972	-0.8577	0.5461
3	1	0.4139	0.2521	0.475	-0.2880	1.1157
	2	0.9359	0.2521	0.003	0.2340	1.6377
	4	0.8462	0.2521	0.010	0.1443	1.5481
	5	0.7801	0.2521	0.022	0.0782	1.4820
4	1	-0.4324	0.2521	0.430	-1.1343	0.2695
	2	0.0896	0.2521	0.997	-0.6123	0.7915
	3	-0.8462	0.2521	0.010	-1.5481	-0.1443
	5	-0.0661	0.2521	0.999	-0.7680	0.6358
5	1	-0.3662	0.2521	0.596	-1.0681	0.3357
	2	0.1558	0.2521	0.972	-0.5461	0.8577
	3	-0.7801	0.2521	0.022	-1.4820	-0.0782
	4	0.0661	0.2521	0.999	-0.6358	0.7680

Table S8: Tukey HSD Post Hoc Comparisons with Adjusted p-values on the activation cluster of Right PGp

(I) Information Level	(J) Information Level	Mean Difference (I-J)	Std. Error	Sig.	95% Confidence Interval	
					Lower Bound	Upper Bound
1	2	0.0944	0.2829	0.997	-0.6931	0.8820
	3	0.0061	0.2829	1.000	-0.7815	0.7936
	4	-0.4818	0.2829	0.437	-1.2693	0.3058
	5	-0.6065	0.2829	0.211	-1.3941	0.1811
2	1	-0.0944	0.2829	0.997	-0.8820	0.6931
	3	-0.0884	0.2829	0.998	-0.8760	0.6992
	4	-0.5762	0.2829	0.257	-1.3638	0.2114
	5	-0.7010	0.2829	0.105	-1.4885	0.0866
3	1	-0.0061	0.2829	1.000	-0.7936	0.7815
	2	0.0884	0.2829	0.998	-0.6992	0.8760
	4	-0.4878	0.2829	0.424	-1.2754	0.2998
	5	-0.6126	0.2829	0.202	-1.4002	0.1750
4	1	0.4818	0.2829	0.437	-0.3058	1.2693
	2	0.5762	0.2829	0.257	-0.2114	1.3638
	3	0.4878	0.2829	0.424	-0.2998	1.2754
	5	-0.1248	0.2829	0.992	-0.9123	0.6628
5	1	0.6065	0.2829	0.211	-0.1811	1.3941
	2	0.7010	0.2829	0.105	-0.0866	1.4885
	3	0.6126	0.2829	0.202	-0.1750	1.4002
	4	0.1248	0.2829	0.992	-0.6628	0.9123

Table S9: Tukey HSD Post Hoc Comparisons with Adjusted p-values on the activation cluster of Right Ph2

(I) Information Level	(J) Information Level	Mean Difference (I-J)	Std. Error	Sig.	95% Confidence Interval	
					Lower Bound	Upper Bound
1	2	0.2874	0.2180	0.681	-0.3196	0.8944
	3	-0.2169	0.2180	0.857	-0.8239	0.3901
	4	-0.1988	0.2180	0.892	-0.8058	0.4082
	5	-0.4497	0.2180	0.246	-1.0567	0.1573
2	1	-0.2874	0.2180	0.681	-0.8944	0.3196
	3	-0.5043	0.2180	0.150	-1.1113	0.1027
	4	-0.4862	0.2180	0.178	-1.0932	0.1208
	5	-0.7371	0.2180	0.009	-1.3441	-0.1301
3	1	0.2169	0.2180	0.857	-0.3901	0.8239
	2	0.5043	0.2180	0.150	-0.1027	1.1113
	4	0.0181	0.2180	1.000	-0.5889	0.6251
	5	-0.2328	0.2180	0.822	-0.8399	0.3742
4	1	0.1988	0.2180	0.892	-0.4082	0.8058
	2	0.4862	0.2180	0.178	-0.1208	1.0932
	3	-0.0181	0.2180	1.000	-0.6251	0.5889
	5	-0.2509	0.2180	0.779	-0.8579	0.3561
5	1	0.4497	0.2180	0.246	-0.1573	1.0567
	2	0.7371	0.2180	0.009	0.1301	1.3441
	3	0.2328	0.2180	0.822	-0.3742	0.8399
	4	0.2509	0.2180	0.779	-0.3561	0.8579

Table S10: Tukey HSD Post Hoc Comparisons with Adjusted p-values on the activation cluster of Left hIP5

(I) Information Level	(J) Information Level	Mean Difference (I-J)	Std. Error	Sig.	95% Confidence Interval	
					Lower Bound	Upper Bound
1	2	0.6086	0.2582	0.137	-0.1101	1.3274
	3	-0.4437	0.2582	0.428	-1.1624	0.2750
	4	0.0697	0.2582	0.999	-0.6491	0.7884
	5	0.0127	0.2582	1.000	-0.7060	0.7315
2	1	-0.6086	0.2582	0.137	-1.3274	0.1101
	3	-1.0523	0.2582	$<$0.001	-1.7710	-0.3336
	4	-0.5390	0.2582	0.234	-1.2577	0.1798
	5	-0.5959	0.2582	0.152	-1.3146	0.1228
3	1	0.4437	0.2582	0.428	-0.2750	1.1624
	2	1.0523	0.2582	$<$0.001	0.3336	1.7710
	4	0.5133	0.2582	0.280	-0.2054	1.2321
	5	0.4564	0.2582	0.399	-0.2623	1.1751
4	1	-0.0697	0.2582	0.999	-0.7884	0.6491
	2	0.5390	0.2582	0.234	-0.1798	1.2577
	3	-0.5133	0.2582	0.280	-1.2321	0.2054
	5	-0.0569	0.2582	0.999	-0.7757	0.6618
5	1	-0.0127	0.2582	1.000	-0.7315	0.7060
	2	0.5959	0.2582	0.152	-0.1228	1.3146
	3	-0.4564	0.2582	0.399	-1.1751	0.2623
	4	0.0569	0.2582	0.999	-0.6618	0.7757

Table S11: Tukey HSD Post Hoc Comparisons with Adjusted p-values on the activation cluster of Left hIP3

(I) Information Level	(J) Information Level	Mean Difference (I-J)	Std. Error	Sig.	95% Confidence Interval	
					Lower Bound	Upper Bound
1	2	0.2914	0.2588	0.793	-0.4292	1.0119
	3	-0.5295	0.2588	0.253	-1.2501	0.1910
	4	0.0576	0.2588	0.999	-0.6630	0.7781
	5	-0.1269	0.2588	0.988	-0.8475	0.5937
2	1	-0.2914	0.2588	0.793	-1.0119	0.4292
	3	-0.8209	0.2588	0.017	-1.5415	-0.1003
	4	-0.2338	0.2588	0.895	-0.9543	0.4868
	5	-0.4183	0.2588	0.491	-1.1388	0.3023
3	1	0.5295	0.2588	0.253	-0.1910	1.2501
	2	0.8209	0.2588	0.017	0.1003	1.5415
	4	0.5871	0.2588	0.165	-0.1334	1.3077
	5	0.4026	0.2588	0.530	-0.3179	1.1232
4	1	-0.0576	0.2588	0.999	-0.7781	0.6630
	2	0.2338	0.2588	0.895	-0.4868	0.9543
	3	-0.5871	0.2588	0.165	-1.3077	0.1334
	5	-0.1845	0.2588	0.953	-0.9051	0.5361
5	1	0.1269	0.2588	0.988	-0.5937	0.8475
	2	0.4183	0.2588	0.491	-0.3023	1.1388
	3	-0.4026	0.2588	0.530	-1.1232	0.3179
	4	0.1845	0.2588	0.953	-0.5361	0.9051

Table S12: Tukey HSD Post Hoc Comparisons with Adjusted p-values on the activation cluster of Left PGp

(I) Information Level	(J) Information Level	Mean Difference (I-J)	Std. Error	Sig.	95% Confidence Interval	
					Lower Bound	Upper Bound
1	2	0.4947	0.2467	0.272	-0.1920	1.1815
	3	-0.3937	0.2467	0.504	-1.0805	0.2930
	4	-0.2055	0.2467	0.920	-0.8922	0.4813
	5	-0.1922	0.2467	0.936	-0.8790	0.4945
2	1	-0.4947	0.2467	0.272	-1.1815	0.1920
	3	-0.8884	0.2467	0.005	-1.5752	-0.2017
	4	-0.7002	0.2467	0.043	-1.3870	-0.0135
	5	-0.6870	0.2467	0.050	-1.3737	-0.0002
3	1	0.3937	0.2467	0.504	-0.2930	1.0805
	2	0.8884	0.2467	0.005	0.2017	1.5752
	4	0.1882	0.2467	0.940	-0.4985	0.8750
	5	0.2015	0.2467	0.925	-0.4853	0.8882
4	1	0.2055	0.2467	0.920	-0.4813	0.8922
	2	0.7002	0.2467	0.043	0.0135	1.3870
	3	-0.1882	0.2467	0.940	-0.8750	0.4985
	5	0.0133	0.2467	1.000	-0.6999	0.7000
5	1	0.1922	0.2467	0.936	-0.4945	0.8790
	2	0.6870	0.2467	0.050	0.0002	1.3737
	3	-0.2015	0.2467	0.925	-0.8882	0.4853
	4	-0.0133	0.2467	1.000	-0.7000	0.6999

Table S13: Tukey HSD Post Hoc Comparisons with Adjusted p-values on the activation cluster of Left FG2
